# Long-term storage (1–4 years) maintains nutritional value of wheat for geese: Metabolic energy and nutrient utilization

**DOI:** 10.1016/j.psj.2025.105851

**Published:** 2025-09-16

**Authors:** Lei Xu, Yonglei Yang, Haiming Yang, Yaqian Liang, Zhi Yang, Zhiyue Wang

**Affiliations:** a College of Animal Science and Technology, Yangzhou University, Yangzhou 225009, China; b Joint International Research Laboratory of Agriculture and Agri-Product Safety of Ministry of Education of China, Yangzhou University, Yangzhou 225009, China

**Keywords:** AA availability, Goose, AME, Nutrient utilization, Stored wheat

## Abstract

We evaluated the nutritional value of wheat stored for 1–4 years in geese through metabolic trials, focusing on metabolizable energy (AME and TME), nutrient composition, amino acid (AA)utilization, and non-starch polysaccharides (**NSP**). A randomized experiment utilized 24 healthy adult male geese (aged 280 days, 4.31 ± 0.25 kg), evenly divided into four groups (six replicates per group, one goose per replicate). Wheat samples of the same variety, origin, and planting method were collected, representing storage durations of 1, 2, 3, and 4 years. Wheat stored for 1–4 years maintained consistently high levels of gross energy (GE), crude protein (CP), AA, and starch, and no mycotoxins were detected. The dry matter, GE, CP, crude fat, total phosphorus, starch, and AA contents remained stable across storage years (*CV* < 5 %). In contrast, acid detergent fiber (ADF), neutral detergent fiber (NDF), mannan, and β-glucan exhibited significant variations (*CV* > 5 %). Notably, geese’s utilization efficiency of mannan, β-glucan, hemicellulose, ADF, NDF, and starch improved linearly with longer storage periods (*P* < 0.05). The apparent metabolizable energy (AME) values for 1-, 2-, 3-, and 4-year-old wheat were 14.12, 14.45, 15.34, and 13.59 MJ/kg (mean: 14.38 MJ/kg), while true metabolizable energy (TME) values were 15.35, 15.54, 16.40, and 14.76 MJ/kg (mean: 15.51 MJ/kg). AME and TME peaked at 3 years of storage but declined at 4 years. In conclusion, wheat without mycotoxin contamination retained nutritional suitability for geese during 1–4 years storage, although a reduction in nutritional value was observed after 4 years.

## Introduction

Global grain production exceeded 2.84 billion tons in 2023/2024, with wheat constituting 28.02 % of the total (FAO, 2024). To stabilize grain prices and ensure supply security against natural disasters and market fluctuations, some countries, such as China maintains strategic wheat reserves. Hu et al. (2023) suggested that wheat stored for more than one year could not maintain a reasonable quality for human food. Annually, substantial quantities of stored wheat—no longer suitable for human consumption—are released for industrial uses, such as alcohol processing and animal feed, due to its cost-effectiveness (Licciardello et al., 2017; Hu et al., 2019). Compared to stored maize, stored wheat offers greater economic advantages (Qiu et al., 2010), making it a promising feed ingredient for geese. However, its nutritional value must be systematically evaluated to optimize its application in goose diets.

The nutritional quality of stored wheat is influenced by multiple factors, including physical conditions (temperature, humidity), biological agents (microflora, arthropods, vertebrates), and technical aspects (storage methods, duration) (Gutiérrez et al., 2008; Li et al., 2022). These factors collectively alter the physicochemical and sensory properties of wheat, potentially compromising its nutritional value. For instance, while four-year-stored corn reduced broiler performance and muscle quality, it did not impair metabolizable energy, crude protein, or starch utilization (Yin et al., 2017). Mycotoxin contamination, a critical concern in both pre- and post-harvest stages (Magan et al., 2010), can further diminish poultry productivity and product quality when contaminated feed is used (Guo et al., 2023). Additionally, wheat’s nutritional variability depends on plant variety and storage duration. Wheat varieties have significant diversity of lipid stability after storage (Wei et al., 2021). Some varieties exhibit changes in apparent metabolizable energy (**AME**) after 0–24 weeks of storage (Pirgozliev et al., 2006), though nitrogen-corrected AME (**AMEn**) in wheat distillers’ dried grains with solubles (DDGS) remains stable during ambient storage, with no adverse effects on broilers (Whiting et al., 2018). The wheat’s nutrient digestibility decreased linearly as storage duration increased from three to 12 month as judged by pigs (Guo et al., 2015). However, little is known about the nutritional value of long-term stored wheat in geese. More research is needed to evaluate the post-harvest storage duration on nutrient digestibility using wheat obtained over a number of years (Guo et al., 2015). Thus, it is necessary to evaluate the wheat’s nutritional value in geese using wheat with controlled conditions, including uniform variety, growing field, and planting methods, to isolate the impact of multiple factors.

This study aimed to assess the nutritional value of wheat stored for 1–4 years in geese through metabolic trials, using wheat samples of identical origin and variety. By analyzing changes in metabolizable energy, nutrient composition, and nutrient utilization, this study provides a comprehensive evaluation of long-term stored wheat as a viable feed ingredient for geese.

## Materials and methods

### Ethics statement

All animal care and experimental procedures complied with the Regulations for the Administration of Affairs Concerning Experimental Animals (People's Republic of China). The protocol was approved by the Animal Care and Use Committee of Yangzhou University (Approval No. 202205209).

### Wheat Sampling and Storage

Wheat samples were harvested from the same variety, planting field, and cultivation method in Yangzhou, Jiangsu Province (32°24′N, 119°25′E; subtropical monsoon climate) across four consecutive years (2018–2021). The samples were stored under standardized conditions at the Yangzhou Academy of Agricultural Sciences, complying with national storage regulations (GB/T 7415-2008 2008). The wheat collected for this study was in good condition and showed no signs of mold contamination. Storage conditions included well-ventilated rooms at ambient temperature.

In 2022, samples representing four storage durations (1, 2, 3, and 4 years) were collected. For each duration, six bags (2.0 kg each) were randomly selected from the granary as one sampling unit, homogenized, and 10.0 kg of composite samples were packaged in plastic bags. These were stored at −20 °C until chemical analysis and metabolic trials.

### Experimental design and feeding management

#### Environmental controls

Water intake was carefully regulated to prevent feather wetting and contamination in geese. Strict hygiene protocols were maintained throughout the trial period.

#### Animals and housing

Twenty-four healthy adult male geese (280 days old; 4.31 ± 0.25 kg body weight) were randomly divided into four treatment groups (*n* = 6 per group, one goose per replicate). Each group received one of four wheat samples (stored for 1, 2, 3, or 4 years). Geese were individually housed in stainless steel metabolic cages (65 × 35 × 24 cm) equipped with fecal collection trays. Prior to the trial, all equipment and facilities were sterilized, and geese underwent routine immunization and deworming.

#### Metabolic trial protocol

The study employed AME and TME bioassay using an enhanced force-feeding technique for geese (Chen et al., 2019), with modifications from Sibbald's (1976) total fecal collection method. The experimental timeline consisted of 7-day adaptation period: geese acclimated to metabolic cages and were trained for force-feeding procedures. 24 h fasting period: to ensure complete digestive tract clearance (Sheng et al., 2006). 48 h collection period (Sheng et al., 2006): force-feeding of 100 g cracked wheat per goose (Wang et al., 2004). Fecal collection at 0–24 h (primary collection) and 24–48 h (endogenous nitrogen loss determination) (Wang et al., 2004; Sheng et al., 2006; Sheng and Li, 2007). Twice daily, the cage trays were cleaned to prevent coprophagy and microbial fermentation.

#### Sample collection and processing

Fecal samples were manually cleaned to remove feathers and debris, immediately treated with 10 % hydrochloric acid for nitrogen fixation, oven-dried at 65 °C to constant weight, moisture-equilibrated at room temperature for 24 h, ground through a 40-mesh sieve, and stored at −20 °C pending analysis.

### Measurements and analytical procedures

#### Sample preparation protocol

All analytical determinations were conducted on uniformly prepared air-dried samples of experimental feed, collected droppings, and endogenous droppings. Prior to analysis, samples were processed following standardized preparation protocols to ensure measurement accuracy.

#### Conventional nutrients

All the conventional nutrients were measured in reference of Ren and Chen (2020) unless otherwise stated. Gross energy (**GE**) content was quantified using an automated oxygen bomb calorimeter (Model Parr 6100, Parr Instrument Company, USA). Measurements were performed in triplicate with benzoic acid as the calibration standard, following the manufacturer's operational guidelines. The Dry matter (**DM**) content was determined by drying representative samples at 105 ± 2 °C for 24 h in a forced-air drying oven (Model DHG-9203A, Shanghai Jipu Electronic Technology Co., China) until constant weight was achieved. The Crude protein (**CP**) content was measured via the Kjeldahl method using an automated nitrogen analyzer (Model Kjeltec 8400, Foss Analytical, Germany), with a nitrogen-to-protein conversion factor of 6.25. The crude fat (**EE**) content was determined through continuous solvent extraction for 8 h using a Soxhlet extraction system (Model FoodALYT RD40, FoodALYT GmbH, Germany) with petroleum ether as the solvent. The Ash content was measured by incinerating samples at 550 ± 10 °C for 6 h in a muffle furnace (Model SSX2-4-10, Nantong Ruifeng Pharmaceutical Machinery Co., China). Nitrogen-free extract (**NFE**) content (%) = Dry matter content - (Ash content + Crude protein content + Crude fat content + Crude fiber content). Total starch content was measured spectrophotometrically (UV-1780, Shimadzu Corporation, Japan) according to AOAC Method 996.11 (McCleary et al., 1997) using a commercial enzymatic assay kit (SKU: 700004350, Megazyme International, Ireland) based on dual-wavelength measurement at 510 nm and 610 nm.

#### Mycotoxin safety

For mycotoxin analysis, 2.0 (±0.05) g stored wheat were used to quantify aflatoxin B₁ (AFB1), zearalenone (ZEA), and deoxynivalenol (DON), respectively, via indirect competitive enzyme-linked immunosorbent assay (ELISA), using commercial kits (Shenzhen Fende Biotechnology Co., Ltd., China) following the respective standards: AFB1 (Kit No. E001001; GB/T 17480-2008 2008), ZEA (Kit No. E002001; GB/T 19540-2004 2004), and DON (Kit No. E003002; Mokhtar et al., 2022). Each kit contained a pre-coated conjugate antigen ELISA plate, horseradish peroxidase (HRP) conjugate, antibody, standards, and supporting reagents. During analysis, mycotoxins in the sample compete with the pre-coated antigen for antibody binding sites after the addition of standard or sample solutions. Following incubation, an enzyme conjugate was added, and color was developed using tetramethylbenzidine (TMB) substrate. The absorbance measured was inversely proportional to the mycotoxin concentration. Quantification was achieved by comparing results to a standard curve.

#### Fiber components

All the fiber components were measured in reference of the details described by Lin and Zhao, 2018 unless otherwise stated. Crude fiber (CF) was determined via Method with intermediate filtration. Neutral detergent fiber (**NDF**) and acid detergent fiber (**ADF**) contents were determined using the ANKOM filter bag technique (Model A2001, ANKOM Technology, USA) with F57 filter bags. The sequential analysis included: NDF extraction using neutral detergent solution with heat-stable α-amylase; Subsequent ADF extraction using acid detergent solution; Acid detergent lignin (**ADL**) was quantified by combusting the ADF residue at 500 °C for 3 h in the muffle furnace. Hemicellulose content is calculated by subtracting the acid detergent fiber (ADF) value from the neutral detergent fiber (NDF) measurement, while cellulose content is determined by subtracting the acid detergent lignin (ADL) value from the ADF measurement. The soluble non-starch polysaccharides (NSP), including xylan, mannans, and β-glucans, which were determined as followings.

The β-glucan content in wheat and gosling excreta was quantified using the Megazyme β-glucan Assay Kit (SKU: 700004269, Megazyme International, Ireland) according to AOAC Method 995.16 (McCleary and Mugford, 1997). Briefly, wheat samples were hydrated, treated with lichenase, hydrolyzed with β-glucosidase, oxidized by glucose oxidase/peroxidase, and measured spectrophotometrically at 510 nm using a UV-1780 spectrophotometer (Shimadzu Corporation, Japan). The contents of xylan and mannan in wheat and excreta were determined using double-antibody one-step sandwich enzyme-linked immunosorbent assay (ELISA, Nakamura et al., 1991; McCartney et al., 2005; Pattathil et al., 2010) kits for xylan (Kit NO. G27635) and mannan (Kit NO. G36251) purchased from Qingdao Yixin Testing Technology Service Co., Ltd. (Qingdao, China). The substrates were colorimetrically developed, respectively, using TMB, which turns blue under peroxidase catalysis and ultimately yellow upon acidification. The color intensity showed a positive correlation with the substrate concentration in samples. Optical density (OD) values were measured at 450 nm wavelength using a microplate reader, with detailed detection and calculations performed according to the manufacturer's instructions provided in the kits.

#### Mineral content

Calcium (Ca) concentration was measured using the (**EDTA**) complexometric titration method as described by Ren and Chen, 2020, with appropriate quality controls. Total phosphorus (**TP**) content was determined spectrophotometrically (Model UV-1780, Shimadzu Corporation, Japan) using the ammonium molybdate method at 700 nm wavelength.

#### Amino acids (AA)

The main amino acids were determined using an automated amino acid analyzer (Model l-8900, Hitachi High-Technologies, Japan) following acid hydrolysis (6 N HCl, 110 °C, 24 h) with norleucine as internal standard.

### Calculation of energy availability and nutrient utilization

The following equations were used to calculate nutrient utilization parameters: Apparent metabolizable energy (AME, MJ/kg) = (E1 − E0) /F1. The true metabolizable energy (TME, MJ/kg) = (E1 − E0 + E2) / F1. In these equations, E1 represents dietary gross energy intake, E0 denotes total fecal energy, and E2 corresponds to the gross energy of endogenous droppings, and FI represents dietary feed intake. The true utilization ratio of nutrients (%) = (nutrient intake mass − fecal nutrient mass + nutrient endogenous loss)/nutrient intake mass × 100 %. The true utilization ratio of **AA** (%) = (AA intake − AA in droppings + endogenous AA)/AA intake × 100 %. The utilization ratio of fiber components indicates their degradation in geese gut.

### Statistical analysis

Raw data were recorded and organized in Microsoft Excel 2019 (Microsoft Corp., Beijing, China). Statistical analyses were performed using SPSS 26.0 (SPSS Inc., Chicago, IL, USA). One-way ANOVA was applied to assess the effect of storage duration (1–4 years) on wheat nutritional parameters. Duncan’s multiple range test was used for post-hoc comparisons of group means. Statistical significance was set at *P* < 0.05. Each treatment group consisted of six replicates (*n* = 6). Values are reported as means and the standard error of the mean (SEM) unless otherwise stated.

## Results

### Physical and chemical stability of wheat with different storage years

#### Physical and nutritional stability of stored wheat

Table 1 shows the physical properties and conventional nutrient contents of wheat stored for 1–4 years. Wheat samples stored for 1–4 years demonstrated stable physical properties, with a mean thousand-seed weight of 47.72 g (coefficient of variation, *CV* = 6.15 %) and bulk density of 776.4 g/L (*CV* = 3.39 %). Key nutritional components, including dry matter (DM: 87.44–87.93 %), gross energy (GE: 16.67–16.84 MJ/kg), starch (52.17–57.15 %), nitrogen-free extract (NFE: 71.38–75.83 %), crude protein (CP: 13.36–14.28 %), crude fat (EE: 1.82–2.00 %), and total phosphorus (TP: 0.18–0.20 %), and exhibited minimal variation across storage durations (*CV* < 5.0 %). In contrast, ash content (1.86–2.10 %) and calcium levels (0.13–0.16 %) displayed higher variability, with CV values of 5.88 % and 9.19 %, respectively.

**Table 1:** Physical properties and conventional nutrient contents of wheat storaged for 1–4 years (air-dried basis).

Items^1^	Storage durations of wheats				Means	*CV*
	1- Year	2-Years	3- Years	4-Years		
Physical variables						
Thousand seeds’ weight, g	49.30	45.99	46.70	48.90	47.72	6.15
Bulk density, g/L	781.6	817.2	756.5	750.2	776.4	3.39
Conventional nutrients, %						
DM	87.44	87.62	87.82	87.93	87.70	0.21
GE, MJ/kg	16.67	16.82	16.86	16.84	16.80	0.57
Starch	57.15	52.17	54.01	54.36	54.42	1.86
NFE	75.83	71.38	71.74	72.82	72.94	2.40
CP	13.37	13.96	13.36	14.28	13.74	2.87
EE	1.93	1.96	2.00	1.82	1.93	3.36
Ash	2.05	1.86	2.13	2.10	2.05	5.88
Ca	0.13	0.14	0.16	0.15	0.15	9.19
TP	0.18	0.19	0.18	0.20	0.19	3.66

1 DM, dry matter; GE, gross energy; NFE, nitrogen-free extract; CP, crude protein; EE, ether extract; Ca, calcium; TP, total protein.

#### Variability in fiber and NSP

Table 2 shows the fiber components and mycotoxins of wheat storage for 1–4 years. Fiber components showed significant variation with storage duration. Neutral detergent fiber (NDF: 16.27–19.92 %), acid detergent fiber (ADF: 3.27–3.79 %), hemicellulose (9.51–12.88 %), and cellulose (2.65–3.16 %) exhibited *CV* values exceeding 5.0 %. Acid detergent lignin (ADL: 52.01–53.32 %) remained stable (*CV* < 1.5 %). Soluble NSP fractions, including xylan (mean = 4.02 %), mannans (1.27 %), and β-glucans (4.57 %), showed negligible variability (*CV* < 1.5 %).

**Table 2:** Fiber components and mycotoxins of wheat storage for 1–4 years (air-dried basis).

Items^1^	Storage durations of wheats				Means	*CV*		Allowable levels 2
	1-Year	2-Years	3-Years	4-Years				
Fiber components, %								
CF	1.76	1.89	1.90	2.11	1.92	2.74		
NDF	16.27	17.34	19.92	18.59	18.03		8.74	
ADF	3.68	3.27	3.56	3.79	3.58	6.27		
Insoluble NSP, %								
Hemicellulose	9.51	9.97	10.67	12.88	10.76	12.04		
Cellulose	2.83	2.75	3.16	2.56	2.82	7.67		
ADL	52.86	53.32	52.79	52.01	52.75	0.89		
Soluble NSP, mg/g								
Xylan	40.34	37.95	41.24	41.24	40.19	3.87		
Mannans	17.37	10.20	14.43	8.37	12.59	32.34		
β-glucans	44.16	34.29	43.64	54.81	44.23	18.97		
Mycotoxins								
AFB1, μg/kg	Neg^3^	Neg	Neg	Neg	\	\		30
ZEN, μg/kg	Neg	Neg	Neg	Neg	\	\		1000
DON, mg/g	Neg	Neg	Neg	Neg	\	\		5

1 CF, crude fiber; NDF, neutral detergent fiber; ADF, acid detergent fiber; ADL, acid detergent lignin; NSP, non-starch polysaccharides; AFB1, Aflatoxin B1; ZEN, Zearalenone; DON, deoxynivalenol.

2 The allowed maximum levels of mycotoxins according to the Chinese Feed Hygiene Standard (GB13078-2017, 2017).

3 Neg, negative. Tested by the ELISA National Standard Method, mycotoxins (AFB1, ZEN, DON) were not detected (value less than 0.01).

#### Mycotoxin safety

Mycotoxin concentrations (AFB1, ZEA, and DON, Table 2) remained below the detection limit and were consistently lower than the maximum allowable levels defined by the Chinese feed hygiene standards.

### Amino acid composition of wheats

Table 3 shows the amino acids content of wheat storage for 1–4 years. All amino acid (AA) contents remained stable across all storage years, with coefficients of variation (*CV*s) lower than 0.50 %. All the *CV*s were lower than 0.10 %, except for Glu and Pro (0.43 % and 0.13 %, respectively). All essential AAs showed high stability (*CV*s < 0.10 %), with arginine (Arg: 0.55 %), leucine (Leu: 0.73 %), and phenylalanine (Phe: 0.56 %) exhibiting the highest mean concentrations (>0.50 %). Among nonessential amino acids, glutamic acid (Glu: 3.59 %), proline (Pro: 1.12 %), aspartate (Asp: 0.60 %), and serine (Ser: 0.60 %) were predominant, each exceeding 0.5 % in mean content.

**Table 3:** The amino acids content of wheat storage for 1–4 years (air-dried basis).

Items	Storage durations of wheats				Means	*CV*
	1- Year	2-Years	3- Years	4-Years		
Essential amino acids, %						
Leucine	0.63	0.73	0.77	0.78	0.73	0.07
Phenylalanine	0.48	0.57	0.60	0.60	0.56	0.05
Arginine	0.48	0.52	0.60	0.59	0.55	0.06
Glycine	0.42	0.47	0.50	0.50	0.47	0.04
Valine	0.38	0.42	0.44	0.44	0.42	0.03
Threonine	0.33	0.36	0.38	0.38	0.36	0.02
Lysine	0.34	0.32	0.36	0.34	0.34	0.02
Isoleucine	0.29	0.33	0.34	0.35	0.33	0.03
Histidine	0.24	0.27	0.29	0.28	0.27	0.02
Nonessential amino acids, %						
Glutamic acid	2.96	3.70	3.81	3.91	3.59	0.43
Proline	1.32	1.03	1.06	1.07	1.12	0.13
Aspartic acid	0.55	0.58	0.65	0.62	0.60	0.04
Serine	0.52	0.60	0.63	0.63	0.60	0.05
Alanine	0.38	0.41	0.43	0.44	0.42	0.03
Tyrosine	0.15	0.16	0.22	0.23	0.19	0.04

### Conventional nutrient utilization

Table 4 shows the utilization of conventional nutrients and energy in wheat stored for 1–4 years. The average true utilization ratios for crude protein (CP), calcium (Ca), and total phosphorus (TP) in 1–4-year-old wheat were 67.37 %, 55.85 %, and 66.24 %, respectively. Apparent utilization ratios varied across nutrient categories: dry matter (DM, 88.38 %), ether extract (EE, 56.46 %), ash (19.48 %), nitrogen-free extract (NFE, 86.39 %), and starch (95.69 %).

**Table 4:** The true utilization of conventional nutrients and energy in wheat stored for 1–4 years (air-dried basis).

Items^1^	Storage durations of wheats				Mean Value	SEM	*P*-values		
	1- Year	2-Years	3- Years	4-Years			Treatments	Linear	Quadratic
DM, %	88.61	88.89	88.29	87.74	88.38	0.23	0.328	0.124	0.363
EE, %	59.34	59.22	57.13	50.16	56.46	1.62	0.146	0.042	0.275
CP, %	66.84	67.13	68.23	67.27	67.37	0.84	0.950	0.765	0.727
Ash, %	15.82	22.70	15.72	17.29	19.48	1.67	0.675	0.923	0.013
Ca, %	55.86	54.77	58.83	53.95	55.85	1.45	0.684	0.902	0.536
TP, %	68.77	68.35	63.93	63.92	66.24	1.37	0.433	0.138	0.941
Starch, %	94.77	95.34	96.51	96.16	95.69	0.30	0.158	0.048	0.427
NFE, %	86.88	86.82	86.47	85.39	86.39	0.45	0.640	0.251	0.586
GE, %	69.04	70.37	70.63	66.23	69.07	0.90	0.303	0.313	0.122
AME, MJ/kg	14.12^ab^	14.45^ab^	15.34^b^	13.59^a^	14.38	0.22	0.022	0.665	0.010
TME, MJ/kg	15.35^ab^	15.54^ab^	16.40^b^	14.76^a^	15.51	0.21	0.038	0.596	0.022

1 DM, dry matter; EE, ether extract; CP, crude protein; Ca, calcium; TP, total protein; NFE, nitrogen-free extract; GE, gross energy; AME, apparent metabolizable energy; TME, true metabolizable energy. ^a-b^ Means within the same row without shared superscripts differ significantly (*P* < 0.05).

### Energy utilization

Energy utilization are shown in Table 4. Wheat stored for 1–4 years exhibited a gross energy (GE) utilization ratio ranging from 66.23 % to 70.63 %, with an overall mean of 69.07 %. Significant differences (*P* < 0.05) were observed in both AME (mean 14.38 MJ/kg) and TME (mean 15.51 MJ/kg) across storage durations. The AME values for 1-, 2-, 3-, and 4-year-old wheat were 14.12, 14.45, 15.34, and 13.59 MJ/kg, respectively, while corresponding TME values measured 15.35, 15.54, 16.40, and 14.76 MJ/kg. Notably, wheat stored for three years demonstrated significantly higher AME and TME compared to four-year-old wheat (*P* < 0.05).

### Fiber and NSP utilization

Table 5 shows the utilization ratio of fiber components in geese fed with wheat stored for 1–4 years. The utilization ratio of fiber components indicates their degradation in geese gut. The geese’s utilization of water-insoluble fiber components (CF, NDF, ADF, and cellulose) and water-soluble NSP (mannans and β-glucans) in wheat increased linearly (*P* < 0.05) with prolonged storage duration. Among all fiber components, the utilization of NDF, ADF, cellulose, xylan, mannans, and β-glucans showed significant differences (*P* < 0.001) between years three to four compared to year one. In contrast, the utilization of ADL remained stable throughout the 1–4 years of storage.

**Table 5:** The true utilization ratio of fiber components in geese fed with wheat storaged for 1–4 years (air-dried basis).

Variables^1^	Storage durations of wheats				Means	SEM	*P*-values		
	1- Year	2-Years	3- Years	4-Years			Treatments	Linear	Quadratic
CF, %	14.54	15.52	18.21	24.93	18.30	1.74	0.135	0.030	0.388
Van Soest fiber									
NDF, %	66.74^a^	66.83^a^	74.34^b^	77.09^b^	71.25	1.16	<0.001	<0.001	0.351
ADF, %	27.77^b^	22.29^a^	35.13^c^	30.73^b^	28.98	1.10	<0.001	<0.001	0.624
ADL, %	77.88	78.85	77.71	76.08	77.63	0.63	0.504	0.217	0.384
Insoluble non-starch polysaccharides (iNSP)									
Hemicellulose, %	71.27^a^	72.16^ab^	76.45^b^	81.09^c^	75.24	1.09	<0.001	0.021	0.032
Cellulose, %	66.62^ab^	61.15^a^	68.67^b^	60.97^a^	64.35	1.12	0.021	0.761	0.231
Soluble non-starch polysaccharides (sNSP)									
Xylan, %	80.43^a^	78.80^a^	89.38^c^	85.43^b^	83.51	1.18	<0.001	0.126	0.394
Mannan, %	57.80^a^	77.53^c^	67.96^b^	74.48^c^	69.44	2.15	<0.001	<0.001	0.059
Glycogen, %	59.73^a^	66.60^ab^	72.20^b^	62.82^a^	65.34	1.65	<0.001	0.038	0.140

1 CF, crude fiber; NDF, neutral detergent fiber; ADF, acid detergent fiber; ADL, acid detergent lignin. ^a-c^ Means within the same row without shared superscripts differ significantly (*P* < 0.05).

### True amino acid availability

Table 6 shows the true amino acid (AA) utilization of wheat stored for 1–4 years. The average true utilization ratios of AA were stable (*P* > 0.05) in geese fed wheat stored for 1 to 4 years ranged from 93.93 % to 94.22 %. The utilization rates of glycine (Gly), alanine (Ala), tyrosine (Tyr), Leucine (Leu), and Phenylalanine (Phe) increased linearly with increasing storage durations (*P* < 0.05), suggesting a time-dependent enhancement in amino acid bioavailability. Among them, storage for 3 to 4 years resulted in higher geese nutritional utility of Gly, Ala, Tyr, as for geese compared to one year of storage (*P* < 0.05).

**Table 6:** The true amino acid (AA) utilization of wheat stored for 1–4 years (air-dried basis).

Items	Storage Durations				Means	SEM	*P*-values		
	1- Year	2-Years	3- Years	4-Years			Treatments	Linear	Quadratic
Essential AAs, %									
Lysine	81.82	82.72	88.06	85.72	84.58	1.14	0.197	0.097	0.459
Threonine	79.58	82.96	83.30	85.07	82.73	1.19	0.463	0.143	0.743
Arginine	86.52	90.17	92.41	90.57	89.92	0.88	0.103	0.058	0.099
Histidine	86.23	90.05	90.39	89.31	88.99	0.73	0.166	0.135	0.091
Valine	84.43	85.61	85.18	85.84	85.26	0.72	0.924	0.598	0.874
Leucine	87.17	90.59	92.21	91.75	90.43	0.82	0.111	0.035	0.205
Isoleucine	82.76	86.99	86.61	87.42	85.95	0.85	0.184	0.075	0.294
Phenylalanine	89.44	92.36	93.57	93.24	92.15	0.64	0.070	0.022	0.157
Glycine	69.28^a^	80.78^b^	80.56^b^	78.31^b^	77.23	1.41	0.001	0.003	0.001
Nonessential AAs, %									
Alanine	71.60^a^	79.91^b^	81.53^b^	81.05^b^	78.52	1.38	0.015	0.006	0.051
Aspartic acid	81.14	85.29	86.25	85.46	84.53	0.88	0.154	0.074	0.146
Glutamic acid	96.12	96.84	96.73	86.50	96.55	0.18	0.548	0.540	0.224
Proline	95.61	95.68	95.49	96.06	95.71	0.20	0.792	0.543	0.558
Serine	85.91	90.26	90.82	90.69	89.42	0.80	0.069	0.029	0.122
Tyrosine	88.90^a^	91.62^ab^	95.61^b^	97.57^b^	93.42	1.21	0.027	0.004	0.839
Average AA	84.43	88.12	89.25	88.30	87.69	0.14	0.876	0.706	0.895

^a-b^ Means within the same row without shared superscripts differ significantly (*P* < 0.05).

## Discussion

### Mycotoxin safety and nutritional content of stored wheat

Mycotoxin-contaminated grain adversely impacts poultry performance (Guo et al., 2023). In our study, the mycotoxin levels observed in wheat stored for 1–4 years—including AFB1, ZEA, and DON toxins—remained well below the detection line and the maximum allowable levels defined by China’s Feed Hygiene Standard (GB 13078-2017, 2017). The absence of significant mycotoxin accumulation in our study is likely attributable to the optimal storage conditions in Yangzhou, Jiangsu Province, where abiotic factors (temperature, moisture) and biotic controls (preservatives, grain ripeness) mitigated fungal proliferation (Magan et al., 2010). A similar outcome was observed when proper storage protocols and optimal climatic conditions were maintained, resulting in preserved wheat quality with minimal insect infestation, fungal growth, and mycotoxin contamination (Meneghetti et al., 2022).

Consistent with the Tables of Feed Composition and Nutritional Value in China (2022), bulk density, conventional nutrients (DM, GE, starch, NFE, CP, EE, and TP), and all the AAs in stored wheat remained stable across storage durations. Their low *CV*s suggest inherent biochemical stability of wheat during four years storage. The biggest variation was observed in thousand seed’s weight, ash, and calcium (Ca) with *CV*s value above 5 %. Given that mineral content exhibit resistance to oxidation and enzymatic degradation, the variations in ash content and calcium levels may primarily result from interannual climatic differences during the growing stage of wheat. Although long-term storage decreases the ratio of amylose to amylopectin (Guraya et al., 2001; Jhan et al., 2020; Hu et al., 2023), the total starch was only slightly (2.8–5.0 %) decreased during one to four years’ storage in our study. Guo et al. (2015) reported that wheat exhibited declining contents of GE, CP, whereas starch level increased during one year’s storage. These findings support the viability of stored wheat as a cost-effective alternative to corn and soybean meal, offering dual economic and food security benefits.

### Fiber and non-starch polysaccharide (NSP) degradation of stored wheat

The degradation patterns of fiber components and NSP in stored wheat revealed distinct temporal dynamics. In our study, NDF, ADF, and hemicellulose exhibited the most pronounced variability, with four-year-old wheat showing the highest hemicellulose content—a clear indicator of storage duration-dependent effects on hemicellulose accumulation. These findings contrast with Guo et al., 2015 observations of declining NSP content alongside increasing NDF and ADF levels during one-year wheat storage, suggesting differential degradation kinetics between short- and long-term storage periods.

Notably, our data demonstrating improved mannan utilization after 2–4 years of storage align with Gutiérrez et al. (2008)'s reported mechanism of enzymatic hydrolysis progressively breaking down mannan polymers during extended storage. Consistent with geese’s utilization of water-insoluble fiber components (CF, NDF, ADF, and cellulose), the utilization of water-soluble NSP (mannans and β-glucans) in wheat also increased linearly with prolonged storage duration. This phenomenon corresponds to established biochemical pathways involving moisture loss and microbial activity in aging grains. As documented by Sanchez et al. (2021) and Shevkani et al. (2017), prolonged storage triggers two synergistic processes: enzymatic degradation of soluble carbohydrates via amylases and cellulases, combined with microbial (fungal/bacterial) decomposition. These mechanisms collectively explain the observed elevation in fiber fractions (NDF/ADF) and dynamic NSP profile alterations across storage durations. In this study, xylan and mannans were measured using the ELISA method. In the future, more accurate techniques such as high-performance liquid chromatography should be employed to monitor the dynamic changes of the soluble NSP.

### Energy and conventional nutrients utilization across storage durations

The utilization patterns of conventional nutrients in wheat exhibited distinct temporal dependencies during prolonged storage. Linear improvements were observed in starch utilization and six amino acids (leucine, phenylalanine, glycine, alanine, serine, and tyrosine) across storage durations extending from one to four years, contrasting with a concurrent linear decline in EE utilization. Short-term storage effects (3-12 months) demonstrated differential dynamics, showing linear reductions in DM, organic matter, EE, and ADF digestibility in pigs, alongside quadratic changes in NDF digestibility (Guo et al., 2015). Notably, wheat stored for three years achieved peak crude protein (CP) and starch utilization, consistent with Anwar et al. (2023)'s findings of enhanced CP digestibility in 2.5-year-old wheat for poultry. These improvements likely reflect structural modifications in wheat proteins, including reduced anti-nutritional factors and increased enzymatic accessibility (Azhar, 2020).

Starch physicochemical transformations during storage further elucidate these utilization patterns. Oxidative stress-induced structural alterations (Jhan et al., 2020) progressively decrease amylose content while increasing amylopectin ratios (Guraya et al., 2001; Jhan et al., 2020), resulting in reduced peak viscosity and gelatinization enthalpy. These changes enhance nutrient absorption in poultry by lowering intestinal content viscosity (Shevkani et al., 2017; Li et al., 2020).

Lipid stability emerged as a critical degradation pathway, with kernel moisture activating germ lipase and lipoxygenase (Marzocchi et al., 2022). This enzymatic cascade triggered lipid hydrolysis and oxidation, evidenced by a 37.46 % increase in malondialdehyde (lipid oxidation marker) during long-term storage (Marzocchi et al., 2022). Inter-varietal differences amplified these effects, showing up to 30-fold variations in hexanal content after 19 weeks of storage (Wei et al., 2021). The observed reduction in ether extract (EE) utilization in our study could potentially be attributed to these enzymatic reactions and more water -insoluble fibers (NDF and hemicellulose) affecting fatty acid stability during grain storage. The reduction of AME and TME in the fourth year may be attributed to the increase of NDF and ADF content (Guo et al., 2015) in stored wheat, and a reduction of EE utilization by geese. These fiber fraction changes, combined with starch retrogradation and protein-lipid interactions, create a multifactorial framework governing nutrient availability shifts across storage durations.

### Practical implications

Key nutrients (DM, GE, CP, EE, TP, AAs, and starch) remained stable in wheat during 1-4 years of storage, while NDF, ADF, mannan, and β-glucan showed significant variation. Geese maintained or improved utilization of conventional nutrients, energy, fiber, non-starch polysaccharides, and true amino acids from stored wheat. The utilization of six components (starch, NDF, ADF, hemicellulose, mannan, glycogen) and specific AAs (leucine, phenylalanine, glycine, alanine, serine, tyrosine) increased linearly with storage time, with peak utilization of starch, ADF, glycogen, alanine, serine, AME, and TME occurring at three years.

In summary, wheat maintains its nutritional value for geese for up to 4 years of storage under suitable environmental conditions, as evidenced by stable physical-chemical properties, metabolic energy, and nutrient bioavailability. However, a significant decline in energy values was observed after four years, indicating ≤ 3 years as the optimal storage duration for maximal feed efficacy. This study focused exclusively on a single wheat cultivar; consequently, future research should incorporate diverse varieties to systematically evaluate how storage duration affects wheat's nutritional composition in geese diets.

## CRediT authorship contribution statement

**Lei Xu:** Conceptualization. **Yonglei Yang:** Data curation. **Haiming Yang:** Conceptualization. **Yaqian Liang:** Data curation. **Zhi Yang:** Writing – review & editing. **Zhiyue Wang:** Funding acquisition.

## Disclosures

The authors declare the following financial interests/personal relationships which may be considered as potential competing interests:

Zhiyue Wang reports financial support was provided by Chinese Ministry of Finance and Ministry of Agriculture and Rural Affairs. Lei Xu reports was provided by Gaoyou Agriculture and Rural Bureau. If there are other authors, they declare that they have no known competing financial interests or personal relationships that could have appeared to influence the work reported in this paper.
